# Role of conventional radiology and MRi defecography of pelvic floor hernias

**DOI:** 10.1186/1471-2482-13-S2-S53

**Published:** 2013-10-08

**Authors:** Alfonso Reginelli, Graziella Di Grezia, Gianluca Gatta, Francesca Iacobellis, Claudia Rossi, Melchiore Giganti, Francesco Coppolino, Luca Brunese

**Affiliations:** 1 Department of Internal and Experimental Medicine, Magrassi-Lanzara, Institute of Radiology, Second University of Naples, Naples, Italy; 2 University of Ferrara, Dipartimento di Scienze Chirurgiche, Ferrara, Italy; 3 University of Palermo, Department of Radiology, Palermo, Italy; 4 Department of Health Science, University of Molise, Campobasso, Italy

**Keywords:** MRI Defecography, Pelvic Floor, Hernias, Elderly patients

## Abstract

**Background:**

Purpose of the study is to define the role of conventional radiology and MRI in the evaluation of pelvic floor hernias in female pelvic floor disorders.

**Methods:**

A MEDLINE and PubMed search was performed for journals before March 2013 with MeSH major terms 'MR Defecography' and 'pelvic floor hernias'.

**Results:**

The prevalence of pelvic floor hernias at conventional radiology was higher if compared with that at MRI. Concerning the hernia content, there were significantly more enteroceles and sigmoidoceles on conventional radiology than on MRI, whereas, in relation to the hernia development modalities, the prevalence of elytroceles, edroceles, and Douglas' hernias at conventional radiology was significantly higher than that at MRI.

**Conclusions:**

MRI shows lower sensitivity than conventional radiology in the detection of pelvic floor hernias development. The less-invasive MRI may have a role in a better evaluation of the entire pelvic anatomy and pelvic organ interaction especially in patients with multicompartmental defects, planned for surgery.

## Introduction

Pelvic floor disorders represent a significant cause of morbidity and reduction in quality of life that appear to be increasing in frequency during the last few years [1]. Pregnancy, multiparity, advanced age, menopause, obesity, connective tissue disorders, smoking, chronic obstructive pulmonary disease, are only some of the risk factors that can rise intra abdominal pressure and cause these disorders [2].

Pelvic floor disorders may be associated, with an incidence ranging from 18% to 45%, to the so-called midline pelvic floor sagittal hernias (MPH) that represent the herniation of the peritoneum and/or peritoneal viscera in the Douglas', Retzius', and retrorectal spaces.

Although anamnestic and physical examination represents the first approach in the evaluation of the patients with pelvic floor dysfunction, the diagnostic limitation of the pelvic examination alone has led to the need to use more direct and comprehensive diagnostic methods [3-6].

Purpose of the study is to define the role of conventional radiology and MRI in the evaluation of pelvic floor hernias.

## Materials and methods

### Subjects

A MEDLINE and PubMed search was performed for journals before March 2013 with MeSH major terms 'MR Defecography' and 'pelvic floor hernias'. Non-English speaking literature was excluded.

### Methods

#### Conventional radiology

Entero-colpo-cysto-defecography (ECCD) is considered the gold standard for the evaluation of the patients with pelvic floor disorders and diagnosis of MPH [7-9]. For this exam no bowel preparation [10-13]. To obtain small-bowel contrast, 1 h before the exam, 200 mL of barium sulfate 60% p/v is administered to each patient. Through a catheter inserted in the bladder, 400 cc of iodine contrast medium (Ultravist, Bayer Schering Pharma, Berlin, Germany) is injected until the patient felt a sensation of fullness. The patient is placed in the left lateral decubitus position, after which 200 cc of barium paste (Prontobario Esofago 113%, barium paste, Bracco, Milan, Italy) was introduced into the rectum. During injector removal, the anal canal is also contrasted. Vagina is contrasted with 25 ml of barium paste. The fluoroscopic table is then tilted upright 90°, and the patient is seated on a radiolucent commode. An anteroposterior radiograph is taken with the patient at rest; after that, five lateral radiographs are taken at rest, during squeezing, pushing, evacuation, and after evacuation (Table 1).

**Table 1:** Conventional Radiology and MRI Defecography technique

	Conventional Radiology	MRI Defecography
Bladder	400 cc of iodine contrast medium	500-700 mL of water per os 10-15 min before

Vagina	25 ml of barium paste	25-30 mL of gadolinium-diethylenetriamine pentaacetic acid

Rectum	200 cc of barium paste	200 mL of a mixture of ultrasonographic gel

Acquisition	AP at rest, during squeezing, pushing, evacuation and after evacuation	TSE T2 ax, TSE T1 sag, TRUEFISP T2 sag during squeezing, pushing, evacuation


#### Dynamic MR defecography

MRI Defecography should be performed on 1.5-T closed magnet using a body-phased-array receiver coil. To ensure an adequate bladder filling, all patients are invited to drink 500-700 ml of water 10-15 min before the examination. The rectum and vagina should be filled with 200 mL and about 25-30 mL [14], respectively, of a mixture of ultrasonographic gel (Ultragel, G.P.S., Bologna, Italy) and gadolinium-diethylenetriamine pentaacetic acid [3] (Table 1). The study protocol includes TSE T2-W axial (matrix, 181x256; slices, 25; thickness, 5 mm; TR/TE, 6,430/114; flip angle, 180°), TSE T1-W sagittal (matrix, 181x256; slices, 25; thickness, 5 mm; TR/TE, 846/11; flip angle, 150°) sequences, and functional dynamic sequences TRUFISP T2-W sagittal, during squeezing, pushing, and evacuation (matrix, 181x256; slices, 1; thickness, 8 mm; TR/TE, 3.75/ 1.6; flip angle, 80°) (Table 2). The MR-D images so obtained then are assembled in cineview in postprocessing. Examination time took about 30 min to complete.

**Table 2:** MRI defecography protocol

	TSE T2 ax	TSE T1 sag	TRUEFISP T2 sag*
Matrix	181x256	181x256	181x256

Slices	25	25	1

Thickness	5 mm	5 mm	8 mm

TR/TE	6.430/114	846/11	3.75/1.6

FA	180°	150°	80°


#### Image analysis

The reference line used for conventional radiology and MRI is the Pubococcygeal line (PCL), extending from the most inferior portion of the symphysis pubis to the tangent of the sacrococcygeal joint.

The diagnosis of descent of the bladder, vagina, and rectum is based on measurement of the vertical

distance between the PCL and the bladder base, the vaginal vault, and the anorectal junction, respectively.

According to Yang's classification [7], the limits of normal descent with maximal strain are 1.0 cm below the PCL for the bladder base, 1.0 cm above for the vaginal cuff or lower end of the cervix, and 2.5 cm below for the rectal area.

#### Pelvic floor hernia classification

**Figure 1:**
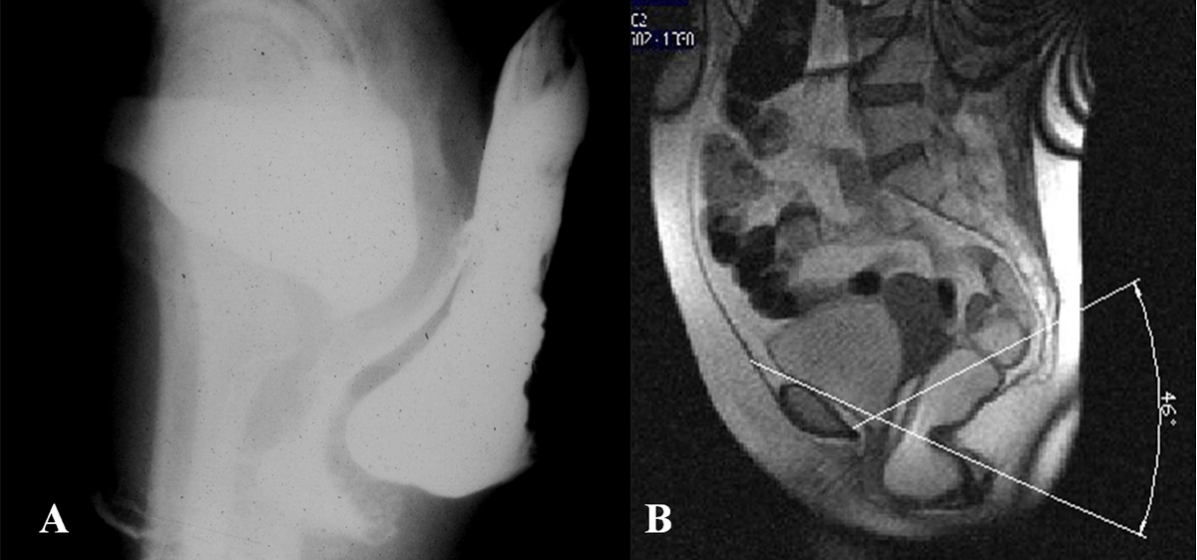
**(a) Rectocele at ECCD defined as an out-pouching of the anterior rectal wall occurring during evacuation or straining, correctly identified also at MR-Defecography(b)**.

Rectocele could be defined as an out-pouching of the anterior rectal wall occurring during evacuation or straining [15-17] (Figure 1a-b).

**Figure 2:**
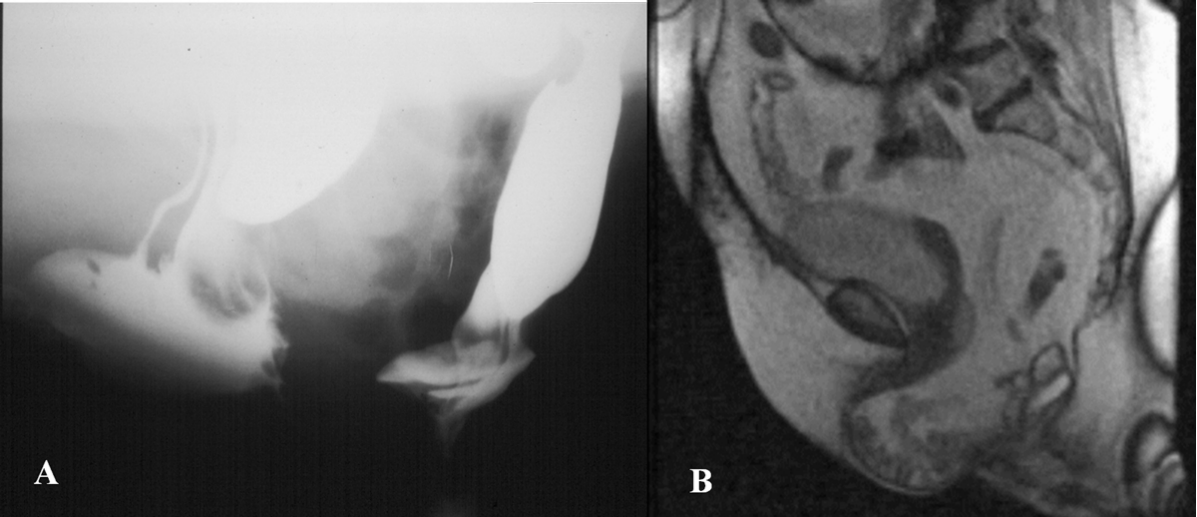
**(a) Enterocele at ECCD: correctly identified also at MR-Defecography(b)**.

Pelvic floor hernias could be classified, basing on the content, into enterocele, omentocele, and sigmoidocele, whereas, according to the hernia development they could be classified as elytrocele, edrocele, retrorectal, and Douglas' and Retzius' hernias [6] (Figure 2a-b).

**Figure 3:**
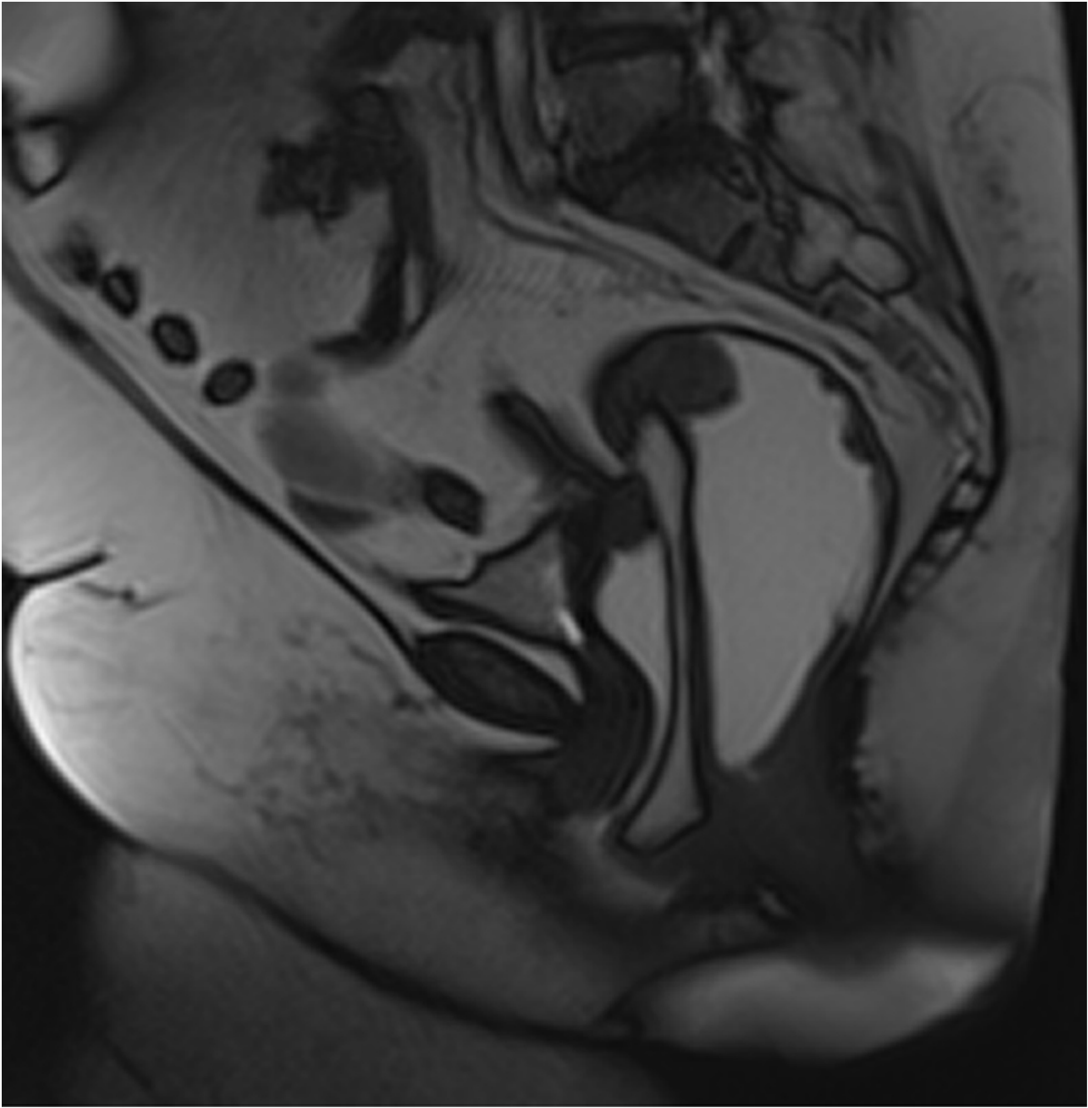
**Omentocele at MR-Defecography: the MR-Defecography clarifies the hernia content as a omentocele**.

Enterocele, sigmoidocele, and omentocele represent the herniation below the proximal (apical) one third of the vagina of the peritoneal sac containing ileal loops, part of the sigmoid, or peritoneal fat, respectively [18-21]. If the small bowel, the peritoneal fat, or the sigmoid colon entered the Retzius' or Douglas' space, they are identified as Retzius' and Douglas' hernias, respectively; if they entered the vaginal fornix posteriorly, causing a complete eversion of the vaginal wall, an elytrocele is recognized (posterior vaginal hernia) [21,22] (Figure 3). In the same way, if they enter the rectum anteriorly, leading to a rectal wall eversion, an edrocele is detected [3,23-25] (Table 3)

**Table 3:** Classification of pelvic floor hernias

Content	Enterocele Omentocele Sigmoidocele
**Development**	Elytrocele (posterior vaginal hernia) Edrocele (anterior rectal hernia) Retrorectal Douglas' hernia Retzius' hernia


#### Conventional radiology diagnosis

**Figure 4:**
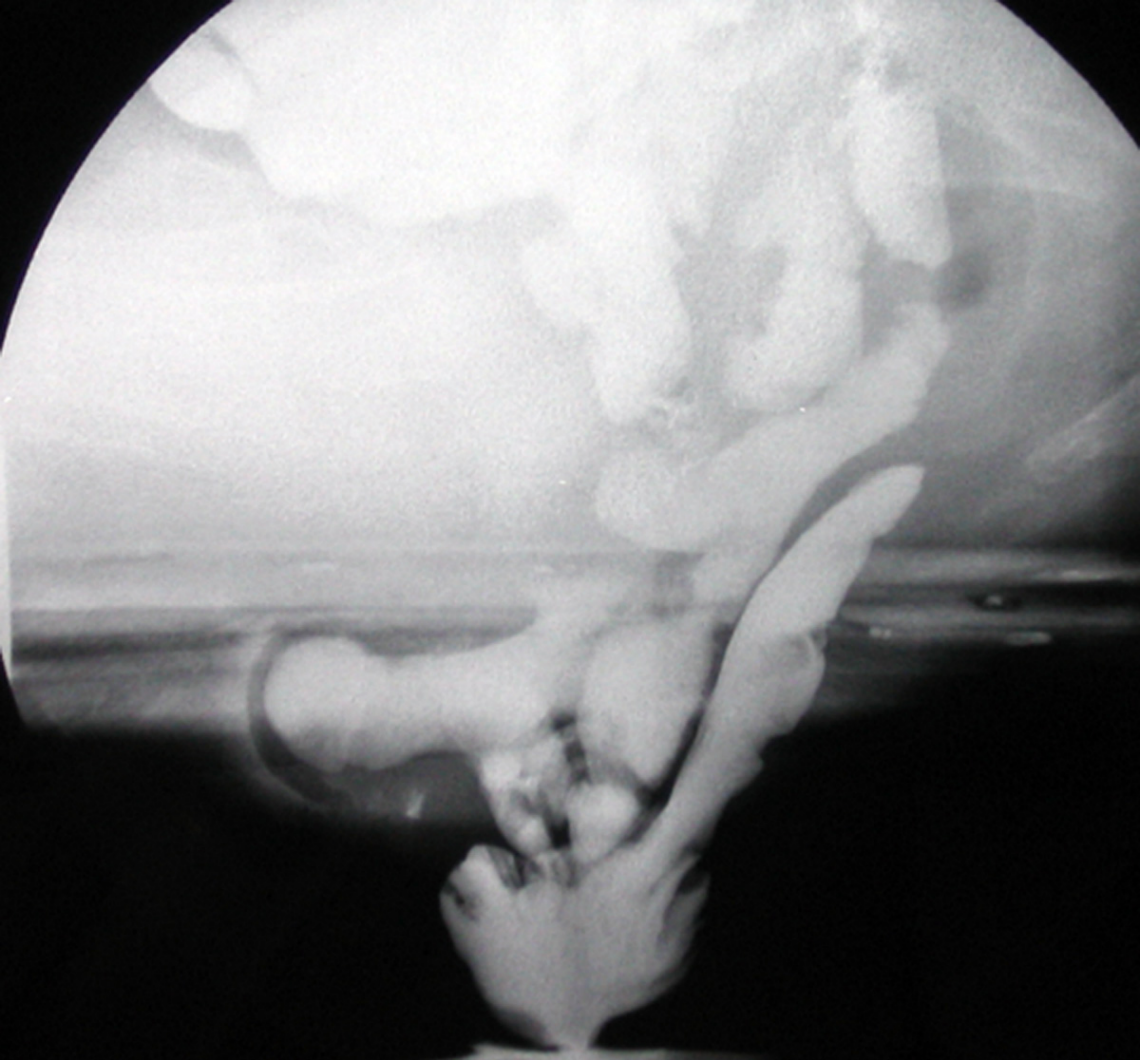
**Elytroceles and Edrocele at ECCD: the small bowel loops enter the vaginal fornix posteriorly with an eversion of the vaginal posterior wall. And the rectum with an eversion of the rectal anterior wall**.

On evaluation of conventional radiology, the diagnosis of an enterocele/ sigmoidocele/omentocele is made if the picture obtained during evacuation compared with that during rest showed an increase in the distance between the vagina and rectum (Figure 4).

This expansion should extend below the PCL reference line and shows a sagittal diameter of more than 2 cm.

Anyway, the distinction between sigmoidocele, enterocele, and omentocele is made basing on the presence of contrasted small bowel in the expanded recto-vaginal space for the enterocele, on the presence of distinguishable bowel gas bubbles without contrast for the sigmoidocele alone, and on the absence of contrasted small bowel and bowel gas bubbles in the expanded recto-vaginal space, for the omentocele.[26-28]

#### Mri defecography diagnosis

On MRI-defecography, the relationship between the lowest point of the peritoneal border line and the PCL should be assessed. A descent of parts of the peritoneal content below this line and the identification of herniated contents allowed the distinction in enterocele, sigmoidocele, and omentocele [8]. The hernias detectable only during pushing and evacuation are considered as "functional hernias."

## Results and discussion

In our experience, the specificity of MRI versus conventional radiology is of a 100%; the sensibility of MR-D in the detection of an omentocele, sigmoidocele, and enterocele is, respectively, 95%, 82%, and 65%, showing an inferior diagnostic capacity if compared with conventional radiology [29,30]. The prevalence of MPH ranged from 38% among all the enrolled patients to 51% in the patients reporting previous hysterectomy. These data are in agreement with the available literature and emphasize the role of previous pelvic surgery in the genesis of MPH [24]. The most frequent hernia is enterocele (70%), followed by sigmoidocele (21%), and omentocele (9%). On the other hand, the most frequent hernia development modality is in Douglas' space (78.9%), whereas the Retzius' and retrorectal hernias represent only occasional findings. The development of the hernias in the posterior vaginal wall or in the anterior rectal wall is observed in 9% and 12% of cases, respectively. Despite their low prevalence, their detection is important in the planning of the correct therapeutic approach. Conventional radiology is currently considered as the gold standard [5,7,8], because is a cost-effective procedure, simple to perform, and widely available [19]; however, it is an invasive procedure, especially if it is performed with four contrast that uses ionizing radiation and visualizes only the lumen of the opacified organs. MRI Defecography was first described by Yang et al. in 1991 [7,31], is a less-invasive imaging modality that allows a multiplanar and multiparametric evaluation of the three pelvic compartments, also visualizing soft tissue, in a single procedure without exposure to ionizing radiation. After this, several studies were performed to compare the diagnostic efficacy of dynamic MRI defecography versus that of conventional radiology in a patient with pelvic floor disorders, with variable results [ 5, 8, 18, 20, 32-34]. In our experience, conventional radiology has higher sensitivity in detecting both the content and the developmnet of pelvic floor hernias if compared with dynamic MRI Defecography. However, the prevalence of enterocele, sigmoidocele, edrocele, elytrocele, and Douglas' hernias at conventional radiology is significantly higher than at MRI Defecography. These findings, in accordance with other authors [5,20], emphasize the role of conventional radiology in the diagnosis of pelvic floor hernias in female pelvic floor disorders, whereas MRI defecography could be more useful to clarify the intra-pelvic interaction of multiple organ prolapse [33] and to better define the pelvic anatomy and functioning in patients planned for surgery [34,35]. Moreover, MRI defecography is a safe, noninvasive exam and free from ionizing radiation[32,36] that is able to correctly define the large bowel loop content of a retrorectal hernia, previously misdiagnosed as an enterocele at coventional radiology [37-40]. The lower sensitivity of MRI Defecography in the detection of pelvic floor hernias may be related to the supine position of the patients [41] and defecation also plays a role by ensuring that intra-abdominal pressure is adequately elevated. A solution on MRI defecography is to repeatedly encourage patients to strain maximally or to monitor intra-abdominal pressure [20].

## Conclusion

In conclusion, MRI defecography shows lower sensitivity than coventional radiology in the detection of pelvic floor hernias. The diagnostic efficacy of conventional radiology is significantly higher than that of MRI Defecography in the detection of both hernia content (enteroceles and sigmoidoceles) and hernia development (Douglas' hernia, elytroceles, and edroceles).

However, the less-invasive MRi defecpgraphy may have a role in a better evaluation of the entire pelvic anatomy and pelvic organ interaction especially in patients with multicompartmental defects, planned for surgery [42].

## Competing interests

The authors declare that they have no competing interests.

## Authors' contributions

AR: conceived the study, analyzed and interpreted the data, drafted the manuscript.

GDG: conceived the study, critically revised the manuscript.

GG: critically revised the manuscript.

FI: critically revised the manuscript.

CR: critically revised the manuscript.

MG: analyzed the data and critically revised the manuscript.

FC: analyzed the data and critically revised the manuscript.

LB: conceived the study, analyzed and interpreted the data, critically revised the manuscript.

All authors read and approved the final manuscript.

## Authors' information

AR: Post-Doctoral Fellow in Radiology at Second University of Naples

GDG: Resident in Radiology Training Program at Second University of Naples

GG: Assistant Professor of Radiology at Second University of Naples

FI: Resident in Radiology Training Program at Second University of Naples

CR: Resident in Radiology Training Program at Second University of Naples

MG: Associate Professor of Radiology, University of Ferrara

FC: PhD Student at University of Palermo

LB: Full Professor of Radiology, University of Molise

## References

[B1] McNevin MS Overview of pelvic floor disorders Surg Clin N Am 2010 90 195 205 20109643 10.1016/j.suc.2009.10.003

[B2] Oom DM Gosselink MP Schouten WR Enterocele diagnosis and treatment Gastroentérol Clin Biol 2009 33 135 7 19200673 10.1016/j.gcb.2009.01.001

[B3] Reginelli A Pezzullo MG Scaglione M Scialpi M Brunese L Grassi R Gastrointestinal disorders in elderly patients Radiol Clin N Am 2008 46 755 771 18922291 10.1016/j.rcl.2008.04.013

[B4] Law YM Fielding JR MRI of pelvic floor disfunction: review AJR 2008 191 S45 S53 19018049 10.2214/AJR.07.7096

[B5] Vanbeckevoort D Van Hoe L Oyen R Ponette E De Ridder D Deprest J Pelvic floor descent in females: comparative study of colpocystodefecography and dynamic fast MR imaging J Magn Reson Imaging 1999 9 373 377 10194705 10.1002/(sici)1522-2586(199903)9:3<373::aid-jmri2>3.0.co;2-h

[B6] Blandino A Rotondo A Danza F Menchi I Pozzi Mucelli R Imaging delle disfunzioni del pavimento pelvico Imaging dell'Apparato Urogenitale Patologia non oncologica 2010 1 Springer

[B7] Yang A Mostwin JL Rosenheim NB Zerhouni EA Pelvic floor descent in women: dynamic evaluation with fast MR Imaging and cinematic display Radiology 1991 179 25 33 2006286 10.1148/radiology.179.1.2006286

[B8] Lienemann A Anthuber A Baron A Kohz P Reiser M Dynamic MR colpocystorectography assessing pelvic-floor descent Eur Radiol 1997 7 1309 17 9377520 10.1007/s003300050294

[B9] Beer-Gabel M Teshler M Schechtman E Zbar AP Dynamic transperineal ultrasound vs. defecography in patients with evacuatory difficulty: a pilot study Int J Colorectal Dis 2004 19 60 67 12761642 10.1007/s00384-003-0508-x

[B10] Cavallo G Salzano A Grassi R Zanatta P Tuccillo M Rectocele in males: clinical, defecographic, and CT study of singular cases Dis Colon Rectum 1991 34 11 964 6 1935474 10.1007/BF02049958

[B11] Rosi G Volterrani L Macarini L Cagini L Cotroneo AR Scialpi M Cough-induced intercostal lung herniation successfully diagnosed with imaging techniques [Ernia polmonare intercostale spontanea tosse-indotta: Diagnosi mediante imaging] Recenti Progressi in Medicina 2012 103 11 523 525 23096744 10.1701/1166.12901

[B12] Scardapane A Rubini G Lorusso F Fonio P Suriano C Giganti M Stabile Ianora AA Role of multidetector CT in the evaluation of large bowel obstruction [Ruolo della TC multidetettore nelle occlusioni del grosso intestino] Recenti Progressi in Medicina 2012 103 11 489 492 23096737 10.1701/1166.12894

[B13] Reginelli A Mandato Y Solazzo A Berritto D Iacobellis F Grassi R Errors in the radiological evaluation of the alimentary tract: part II Semin Ultrasound CT MR 2012 33 4 308 17 22824121 10.1053/j.sult.2012.01.016

[B14] Grassi R Lombardi G Reginelli A Capasso F Romano F Floriani I Colacurci N Coccygeal movement: assessment with dynamic MRI Eur J Radiol 2007 61 473 9 17224255 10.1016/j.ejrad.2006.07.029

[B15] Healy JC Halligan S Reznek RH Watson S Bartram CI Phillips R Armstrong P Dynamic MR imaging compared with evacuation proctography when evaluating anorectal configuration and pelvic floor movement AJR Am J Roentgenol 1997 169 775 9 9275895 10.2214/ajr.169.3.9275895

[B16] Kelvin FM Maglinte DDT Hornback JA Benson JT Pelvic prolapse: assessment with evacuation proctography (defecography) Radiology 1992 184 547 551 1620863 10.1148/radiology.184.2.1620863

[B17] Cl B Tumbull GK Lennard-Jones JE Evacuation proctography: an investigation of rectal expulsion in 20 subjects without defecation disturbance Gastrointest Radiol 1988 3 72 80 10.1007/BF01889028 3350272

[B18] Kelvin FM Maglinte DDT Hale DS Benson JT Female pelvic organ prolapse: a comparison of triphasic dynamic MR imaging and triphasic fluoroscopic cystocolpoproctography AJR Am J Roentgenol 2000 174 1 81 8 10628459 10.2214/ajr.174.1.1740081

[B19] Faccioli N Comai A Mainardi P Perandini S Farah M Pozzi-Mucelli R Defecography: a practical approach Diagn Interv Radiol 2010 16 209 216 20108205 10.4261/1305-3825.DIR.2584-09.1

[B20] Pannu HK Scatarige JC Eng J Comparison of supine magnetic resonance imaging with and without rectal contrast to fluoroscopic cystocolpoproctography for the diagnosis of pelvic organ prolapse J Comput Assist Tomogr 2009 33 125 130 19188799 10.1097/RCT.0b013e318161d739

[B21] Brubaker L Heit MH Radiology of the pelvic floor Clin Obstet Gynecol 1993 36 952 959 8293595 10.1097/00003081-199312000-00019

[B22] Maillard E Henry L Mion F Barth X Tissot E Mellier G Damon H Elytrocele with and without a history of hysterectomy (303 defecography studies) Gastroentérol Clin Biol 2008 32 953 9 18774666 10.1016/j.gcb.2008.04.036

[B23] Dodi G "Colonproctologia ambulatoriale:trattatto per chirurghi, gastroenterologi e madici pratici" ed Piccin 1994

[B24] Guglielmi G Schiavon F Cammarota T Radiologia geriatrica Springer 2006

[B25] Cordiano C D'Amico DF Manuale di Chirurgia d'urgenza Piccin 1981

[B26] Reginelli A Mandato Y Cavaliere C Pizza NL Russo A Cappabianca S Brunese L Rotondo A Grassi R Three-dimensional anal endosonography in depicting anal-canal anatomy [Rappresentazione anatomica del canale anale con ultrasonografia (US) endoanale 3D] (2012) Radiologia Medica 117 5 759 771 10.1007/s11547-011-0768-4 22228126

[B27] Mandato Y Reginelli A Galasso R Iacobellis F Berritto D Cappabianca S Errors in the Radiological Evaluation of the Alimentary Tract: Part I (2012) Seminars in Ultrasound, CT and MRI 33 4 300 307 10.1053/j.sult.2012.01.011 22824120

[B28] Grassi R Lombardi G Reginelli A Capasso F Romano F Floriani I Colacurci N Coccygeal movement: Assessment with dynamic MRI (2007) European Journal of Radiology 61 3 473 479 10.1016/j.ejrad.2006.07.029 17224255

[B29] Cappabianca S Reginelli A Iacobellis F Granata V Urciuoli L Alabiso ME Di Grezia G Marano I Gatta G Grassi R Dynamic MRI defecography vs enterocolpocystodefecography in the evaluation of midline pelvic floor hernias in female pelvic floor disorders Int J Colorectal Dis 2011 26 1191 1196 21538053 10.1007/s00384-011-1218-4

[B30] Sung VW Hampton BS Epidemiology of pelvic floor dysfunction Obstet Gynecol Clin N Am 2009 36 421 43 10.1016/j.ogc.2009.08.002 19932408

[B31] Elshazly WG El Nekady Ael A Hassan H Role of dynamic magnetic resonance imaging in management of obstructed defecation case series Int J Surg 2010 8 274 82 20219700 10.1016/j.ijsu.2010.02.008

[B32] Torricelli P Pecchi A Caruso Lombardi A Vetruccio E Vetruccio S Romagnoli R Magnetic resonance imaging in evaluating functional disorders of female pelvic floor Radiol Med 2002 103 488 500 12207184

[B33] Rentsch M Paetzel Ch Lenhart M Feuerbach S Jauch KW Furst A Dynamic magnetic resonance imaging defecography: a diagnostic alternative in the assessment of pelvic floor disorders in proctology Dis Colon Rectum 2001 44 999 1007 11496081 10.1007/BF02235489

[B34] Matsuoka H Wexner SD Desai MB Nakamura T Nogueras JJ Weiss EG Adami C Billotti VL A comparison between dynamic pelvic magnetic resonance imaging and videoproctography in patients with constipation Dis Colon Rectum 2001 44 571 576 11330585 10.1007/BF02234331

[B35] Goei R Kemerink G Radiation dose in defecography Radiology 1990 176 137 139 2353082 10.1148/radiology.176.1.2353082

[B36] Beer-Gabel M Assoulin Y Amitai M Bardan E A comparison of dynamic transperineal ultrasound (DTP-US) with dynamic evacuation proctography (DEP) in the diagnosis of cul de sac hernia (enterocele) in patients with evacuatory dysfunction Int J Colorectal Dis 2008 23 513 19 18256847 10.1007/s00384-008-0440-1

[B37] Russo S Lo Re G Galia M Reginelli A Lo Greco V D'Agostino T La Tona G Coppolino F Grassi R Midiri M Lagalla R Videofluorography swallow study of patients with systemic sclerosis [Studio videofluorografico della deglutizione in pazienti affetti da sclerodermia sistemica] (2009) Radiologia Medica 114 6 948 959 10.1007/s11547-009-0416-4 19562267

[B38] Krokidis M Orgera G Rossi M Matteoli M Hatzidakis A Interventional radiology in the management of benign biliary stenoses, biliary leaks and fistulas: a pictorial review Insights Imaging 2013 4 1 77 84 23180415 10.1007/s13244-012-0200-1 PMC3579997

[B39] Truta B Allen BA Conrad PG Weinberg V Miller GA Pomponio R Lipton LR Guerra G Tomlinson IP Sleisenger MH Kim YS Terdiman JP A comparison of the phenotype and genotype in adenomatous polyposis patients with and without a family history Fam Cancer 2005 4 2 127 33 15951963 10.1007/s10689-004-5814-0

[B40] Thirlwell C Howarth KM Segditsas S Guerra G Thomas HJ Phillips RK Talbot IC Gorman M Novelli MR Sieber OM Tomlinson IP Investigation of pathogenic mechanisms in multiple colorectal adenoma patients without germline APC or MYH/MUTYH mutations Br J Cancer 2007 96 11 1729 34 17505512 10.1038/sj.bjc.6603789 PMC2359923

[B41] Bertschinger KM Hetzer FH Roos JE Treiber K Marincek B Hilfiker PR Dynamic MR imaging of the pelvic floor performed with patient sitting in an open-magnet unit versus with patient supine in a closed-magnet unit Radiology 2002 223 501 8 11997560 10.1148/radiol.2232010665

[B42] Pescatori M Zbar AP Reintervention after complicated or failed STARR procedure Int J Colorectal Dis 2009 24 87 95 18696087 10.1007/s00384-008-0556-3

